# Unroofed Coronary Sinus in a Patient With Prior Surgical Closure of Secundum Atrial Septal Defect

**DOI:** 10.7759/cureus.19350

**Published:** 2021-11-08

**Authors:** Subrahmanya Murti Velamakanni, Gajanan Khadkikar, Shivam S Panchal, Aman Patel, Bhupesh R Shah

**Affiliations:** 1 Cardiology, Smt. Nathiba Hargovandas Lakhmichand Municipal Medical College, Ahmedabad, IND; 2 Internal Medicine, Smt. Nathiba Hargovandas Lakhmichand Municipal Medical College, Ahmedabad, IND

**Keywords:** persistent left superior venacava, ostium secundum atrial septal defect, transesophageal echocardiography, unroofed coronary sinus, atrial septal defect

## Abstract

An unroofed coronary sinus is an uncommon congenital cardiac anomaly. It leads to a left to right shunt like an atrial septal defect (ASD) and comprises <1% of all ASDs. It can also additionally create a pathway for paradoxical embolization to the brain and other attendant complications. Here, we present the case of an asymptomatic 40-year-old-male with a history of prior surgical closure of an ostium secundum ASD who was referred for preoperative evaluation for non-cardiac surgery. An unroofed coronary sinus with persistent left superior vena cava (PLSVC) was suspected on transthoracic echocardiography and confirmed by transesophageal echocardiography.

## Introduction

An unroofed coronary sinus (CS) is a rare congenital anomaly. It is usually associated with a persistent left superior vena cava (PLSVC). The PLSVC drains to the coronary sinus (CS) that lies in the atrioventricular groove. Due to a defect in the roof of the CS, communication is created from the CS to the left atrium (LA), thereby an indirect communication from the right atrium (RA) to LA is created. This behaves like an atrial septal defect (ASD) leading to right heart overload and pulmonary arterial hypertension. CS ASDs are rare and known to comprise less than one percent of all ASDs [1]. Diagnosis is usually difficult to make on transthoracic echocardiography and requires multimodality imaging like cardiac computed tomography (CT) or magnetic resonance imaging (MRI). A diagnosis can also be made on transesophageal echocardiography and contrast echocardiography via left upper limb access [2]. Multiple secundum ASDs in the same patient are often documented, but a different type of ASD in the same patient is uncommon. Here, we present the case of a 40-year-old male who had undergone prior surgical closure of a secundum ASD 15 years back who was incidentally diagnosed with an unroofed coronary sinus.

## Case presentation

**Figure 1:**
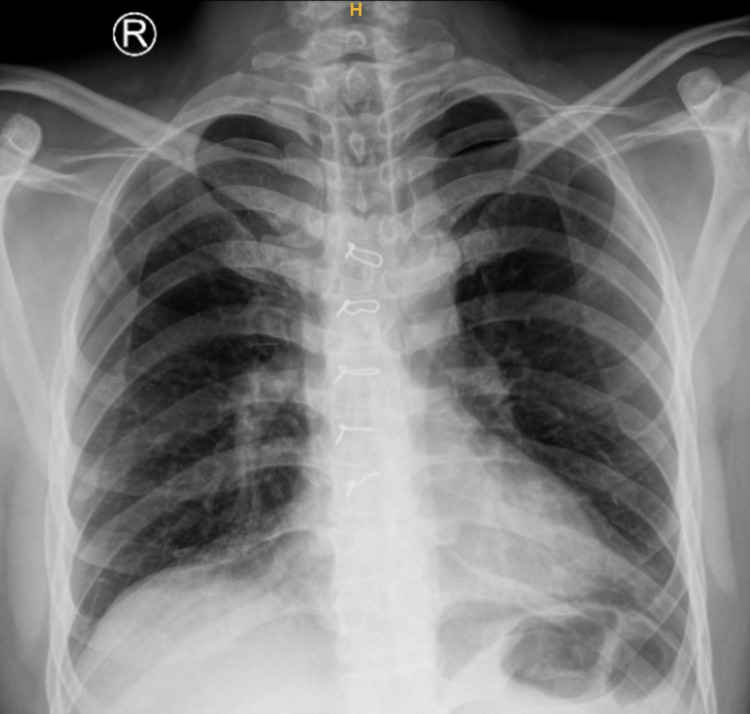
X-ray chest PA view. Prior surgical sternal wires are apparent. PA: posteroanterior.

**Figure 2:**
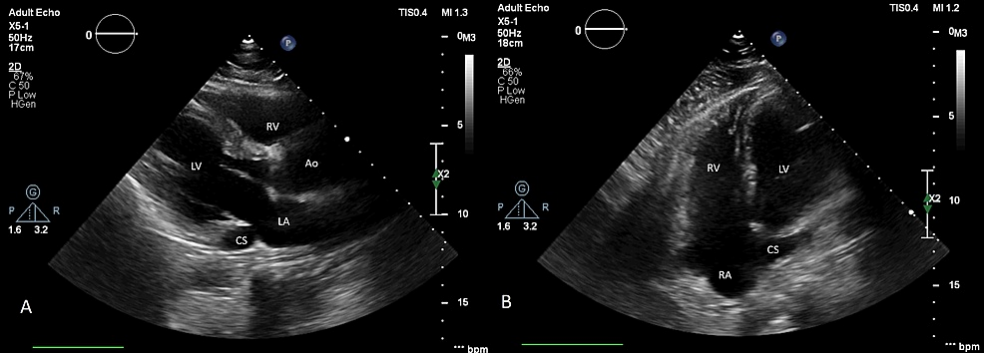
2D transthoracic echocardiographic images: (A) parasternal long-axis view showing a dilated CS; (B) foreshortened apical view showing a dilated CS. 2D: two-dimensional, Ao: aorta, CS: coronary sinus, LA: left atrium, LV: left ventricle, RA: right atrium, RV: right ventricle.

A 40-year-old male with a history of surgical closure of secundum type of atrial septal defect (ASD) 15 years back was referred for cardiac evaluation before elective surgery. Physical examination and electrocardiogram (ECG) were normal. X-ray chest was unremarkable except for prior surgical sternal wires (Figure 1). Transthoracic echocardiography showed situs solitus, levocardia, normal biventricular size, and function. There was no pulmonary arterial hypertension. The coronary sinus (CS) was dilated with suspicion of unroofing along with a persistent left superior vena cava (PLSVC) (Figure 2, Video 1).

**Video 1:** Transthoracic echocardiography showing a parasternal long axis and an apical foreshortened view demonstrating the dilated coronary sinus.

**Figure 3:**
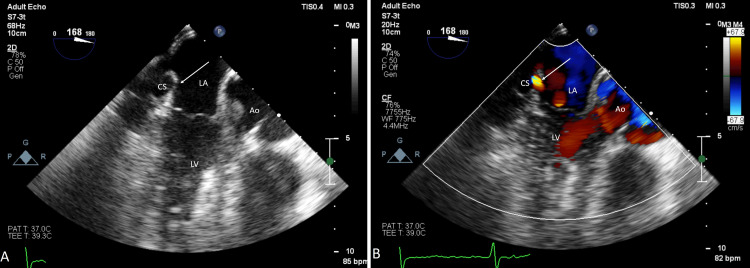
2D transesophageal echocardiographic images: (A) modified view showing the CS. White arrow showing defect on the roof of the CS; (B) view with color Doppler. White arrow shows color flow across the defect on the roof of the CS. 2D: two-dimensional, Ao: aorta, CS: coronary sinus, LA: left atrium, LV: left ventricle.

**Figure 4:**
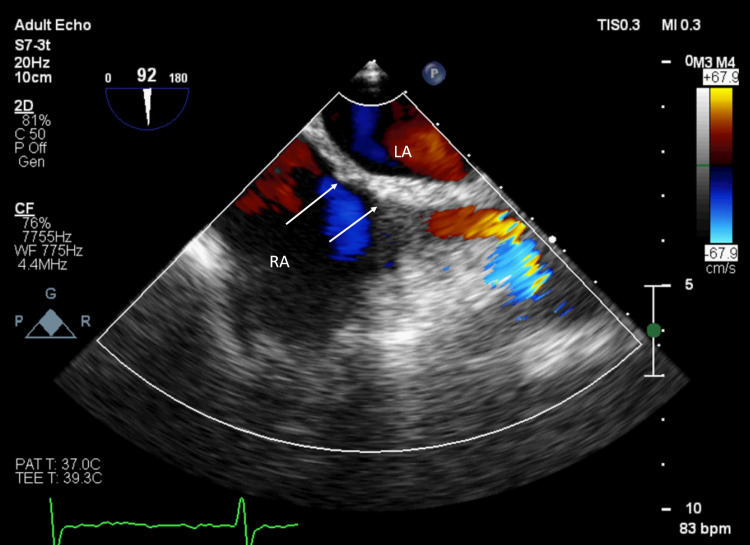
Transesophageal bicaval view. White arrows show an intact patch across the secundum atrial septal defect. LA: left atrium, RA: right atrium.

On transesophageal echocardiography, the coronary sinus was dilated with partial unroofing in the middle part (Figure 3, Video 2). Patch across the interatrial septum in secundum location was intact with no flow across (Figure 4, Video 3). 

**Video 2:** Showing a modified transesophageal echocardiography view at 160 degrees with color Doppler demonstrating a defect in the roof of the coronary sinus.

**Video 3:** Showing transesophageal bicaval view demonstrating intact patch across secundum location.

## Discussion

An unroofed CS has been classified as per the Kirklin and Barratt-Boyes classification into four types [3]. The present case represents a type III defect as there is only partial unroofing in the middle portion. An unroofed CS is an easily corrected defect by surgery. It is known to be associated with other congenital cardiac anomalies like anomalous venous return and heterotaxy.

The diagnosis of an unroofed CS is rarely made only by transthoracic echocardiography alone. On transthoracic echocardiography, an unroofed CS may be suspected by the presence of a dilated CS in the parasternal long-axis view and foreshortened apical view. Additionally, a PLSVC may be sought for in a modified suprasternal view with the transducer oriented slightly to the left of the suprasternal space. Confirmation of the defect usually requires multimodality imaging like cardiac CT or cardiac MRI. A useful alternative is contrast echocardiography with agitated saline via left upper limb access wherein filling of the CS followed by the LA before the RA is seen [2]. Another imaging modality is transesophageal echocardiography, as was done in this case. A study of 20 patients of unroofed CS demonstrated that transesophageal echocardiography, particularly with real-time three-dimensional (3D) imaging is as sensitive as cardiac catheterization [4].

It is also notable that in the present case, there was no pulmonary hypertension or evidence of right-sided volume overload, indicating the fact that the defect was not hemodynamically significant. The patient was advised yearly transthoracic echocardiography and is under regular follow-up.

## Conclusions

Unroofed CS is a rare congenital cardiac defect and requires multimodality imaging for diagnosis. Transesophageal echocardiography can be a useful modality for diagnosis with an excellent image quality, particularly with real-time 3D imaging.
